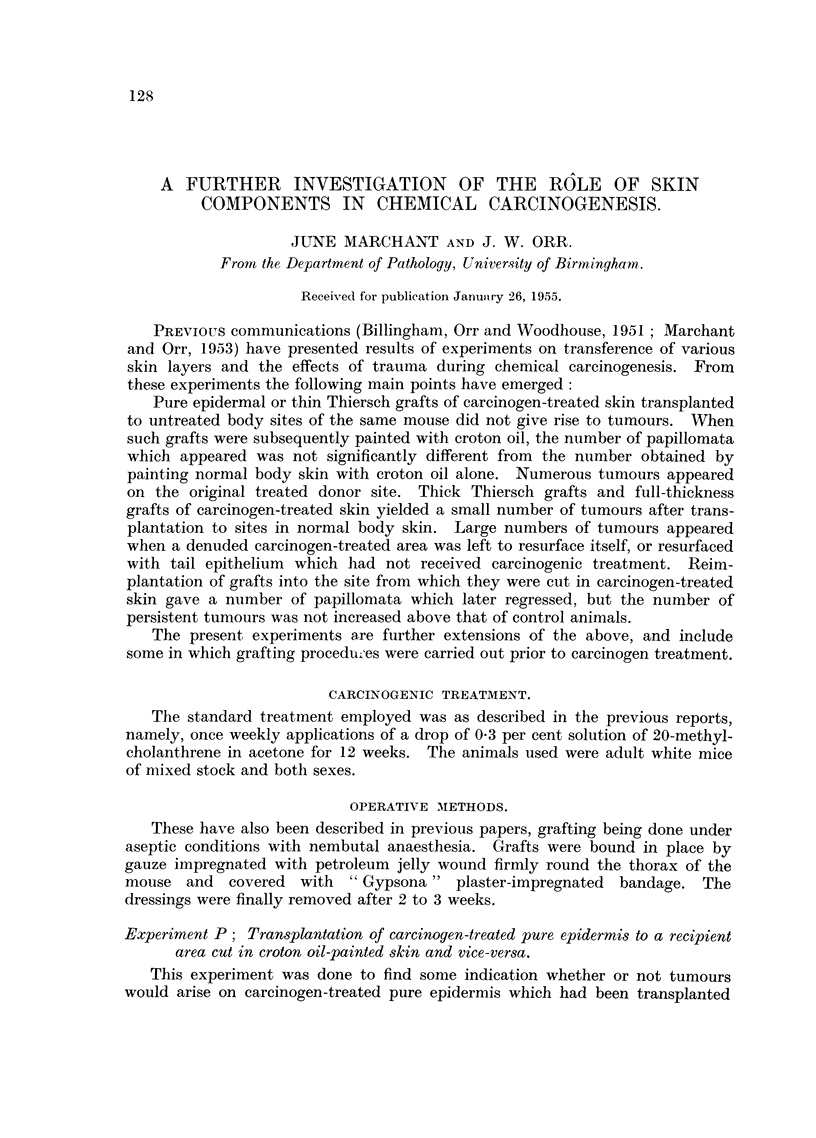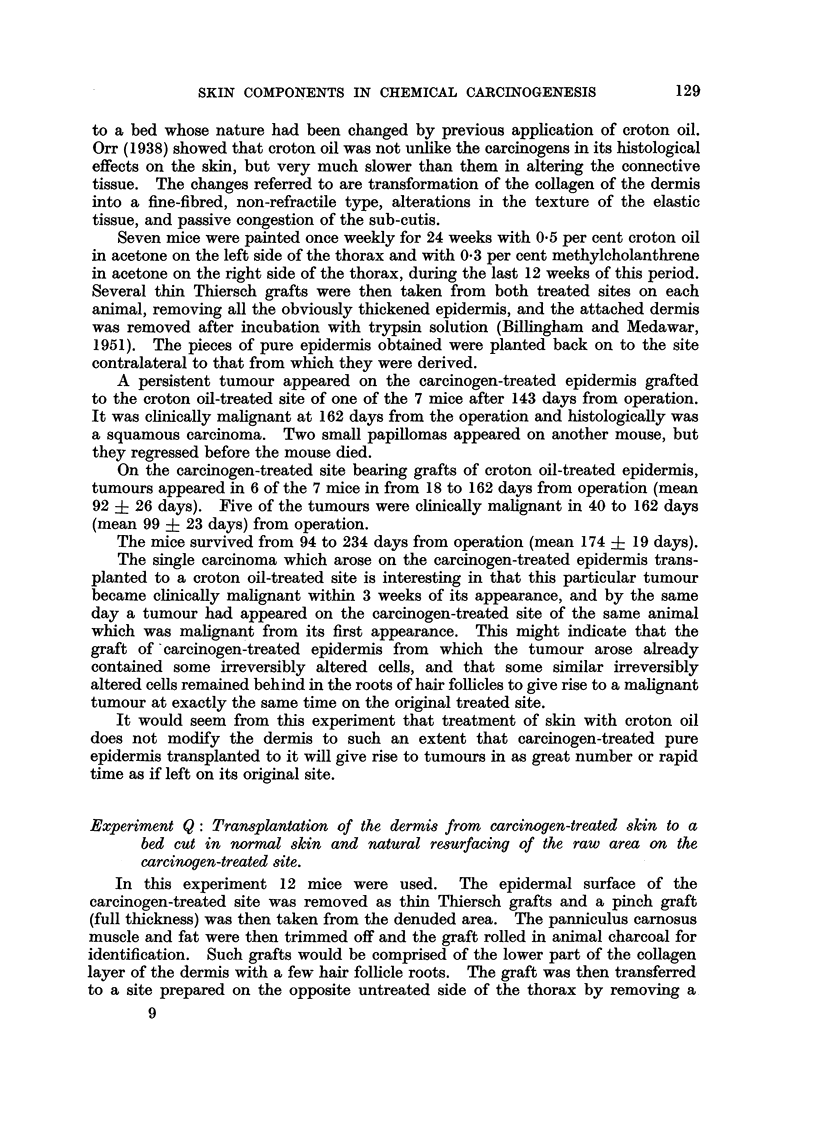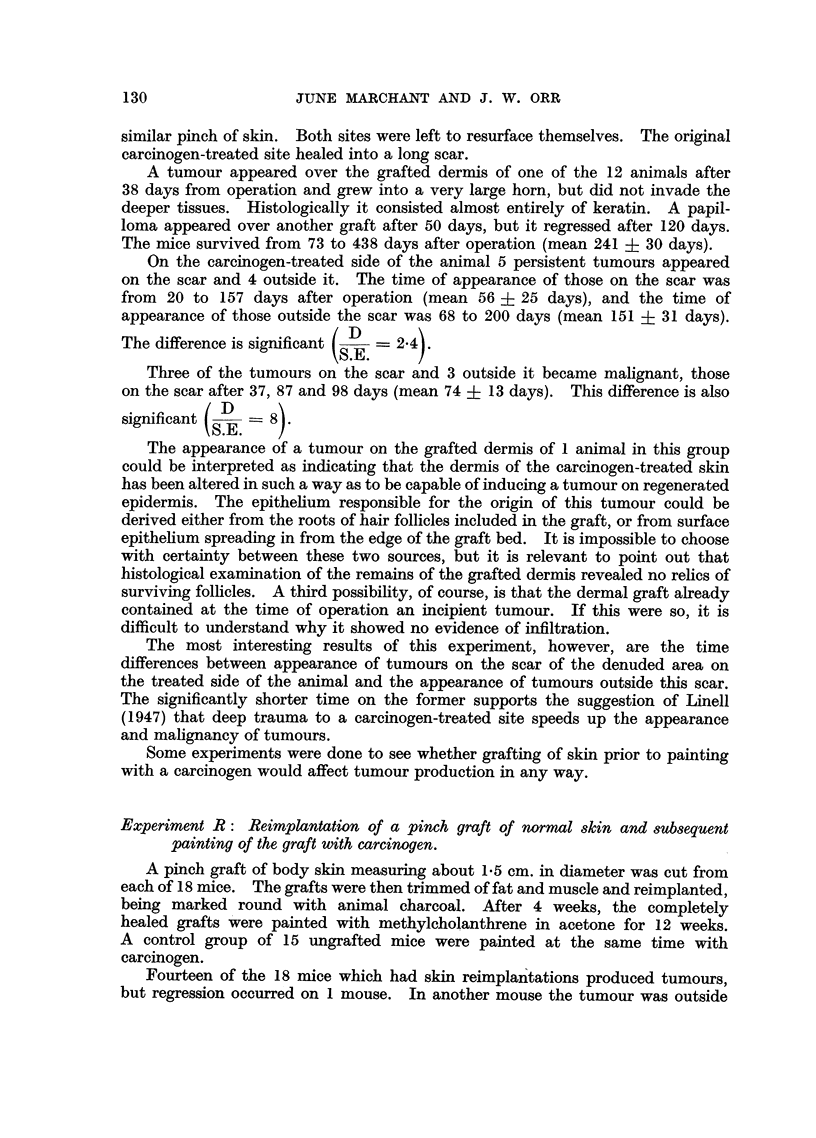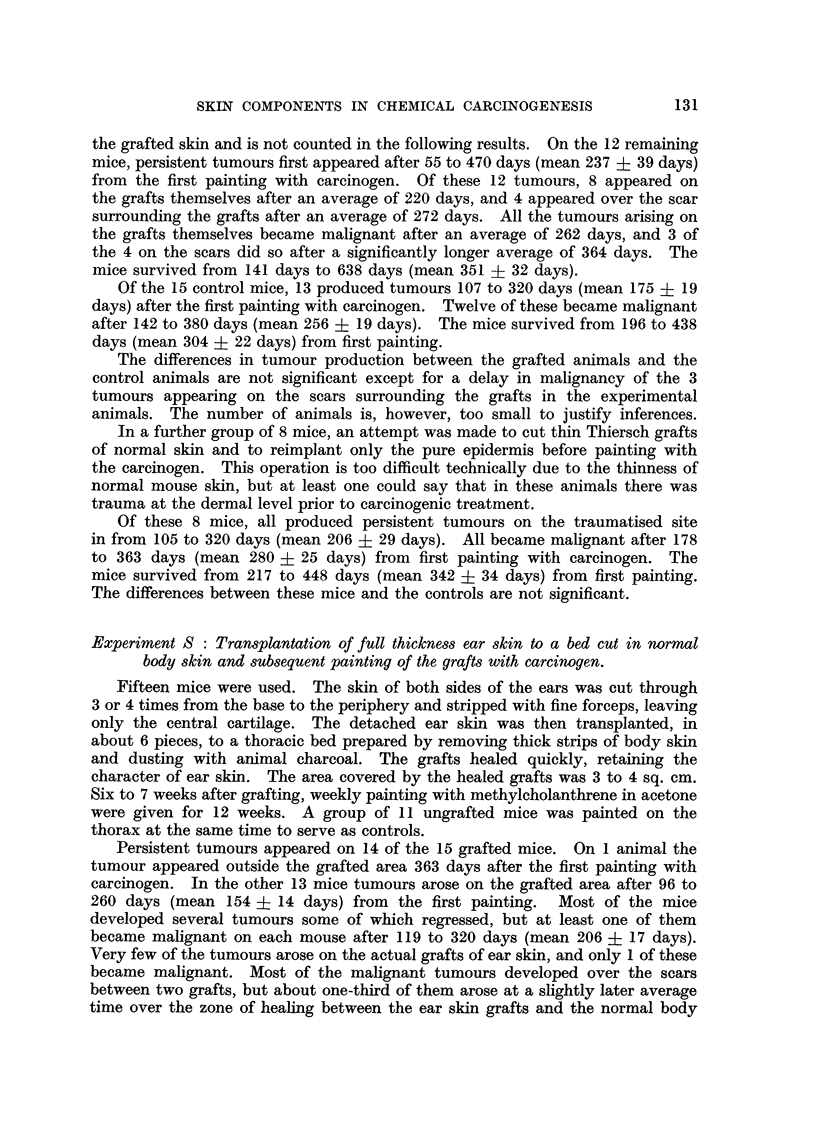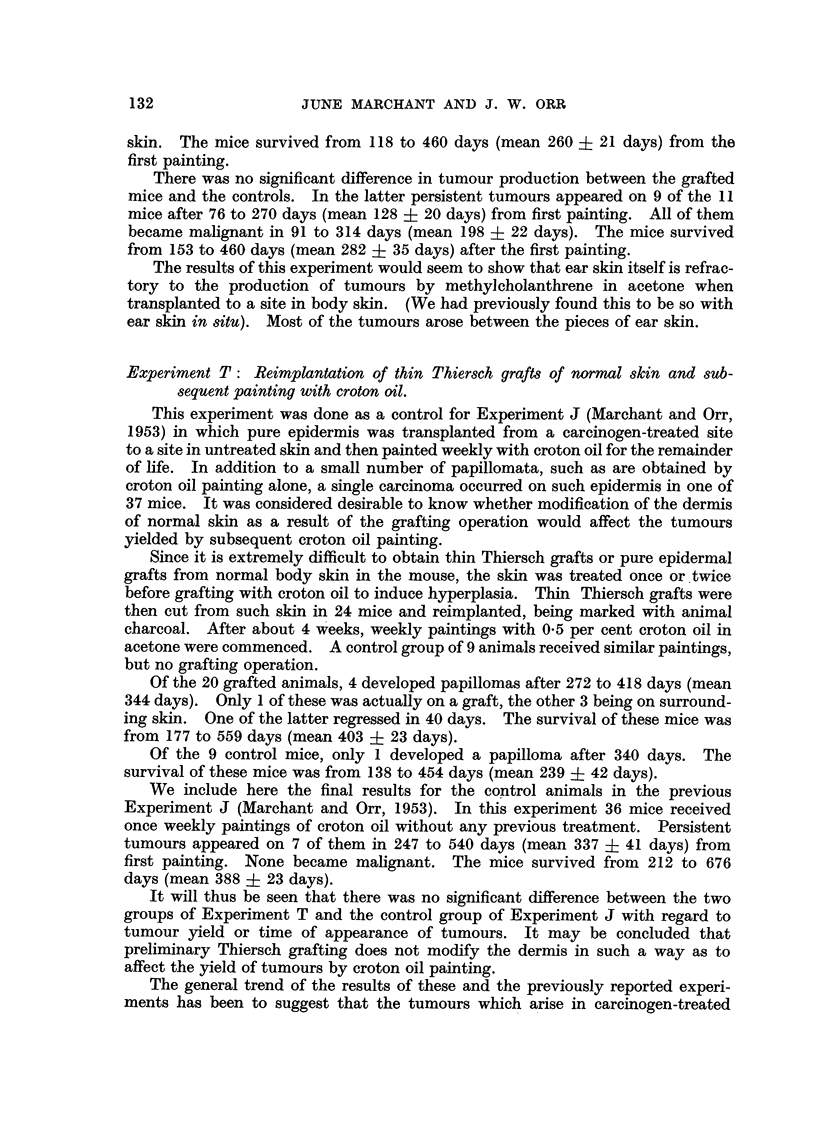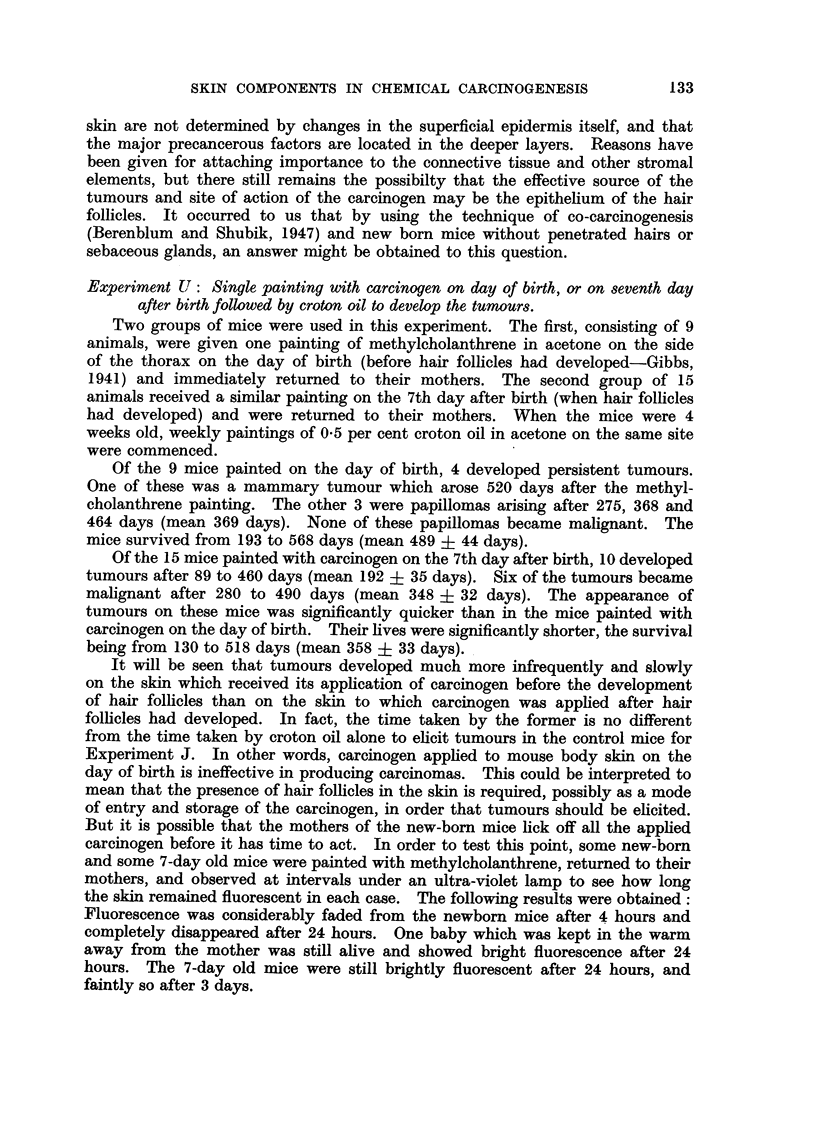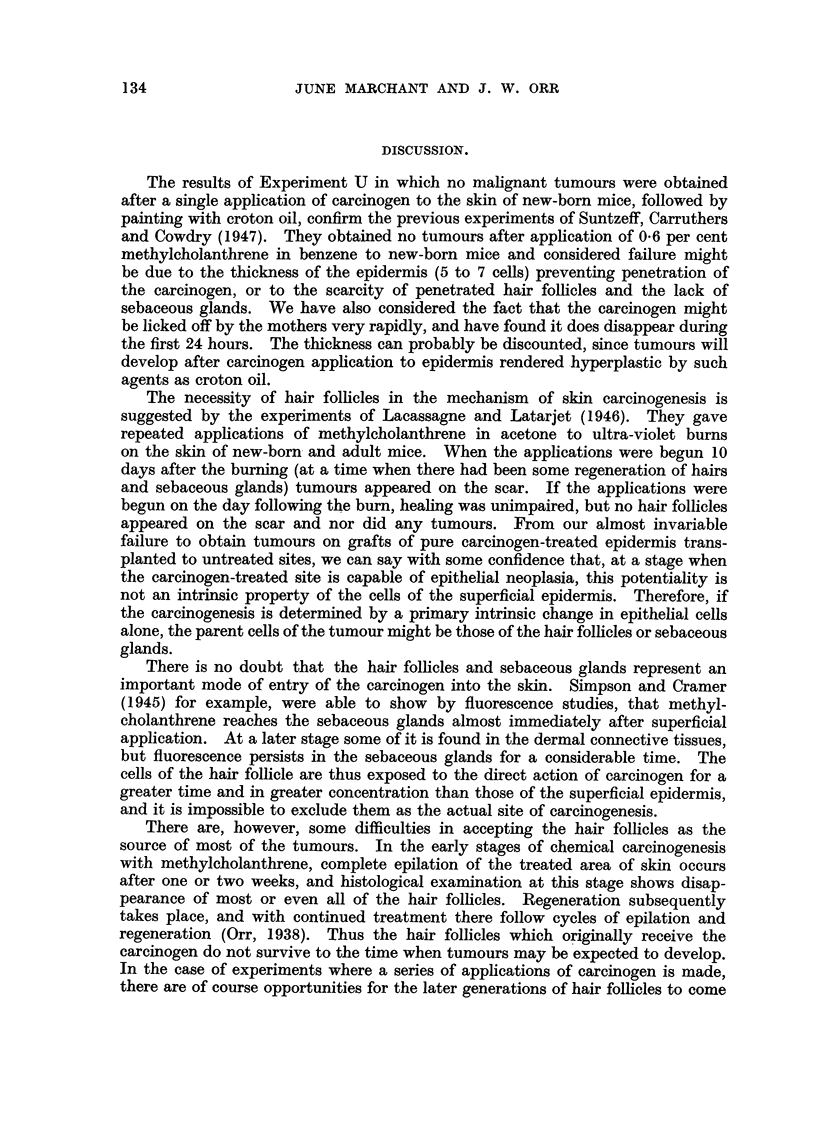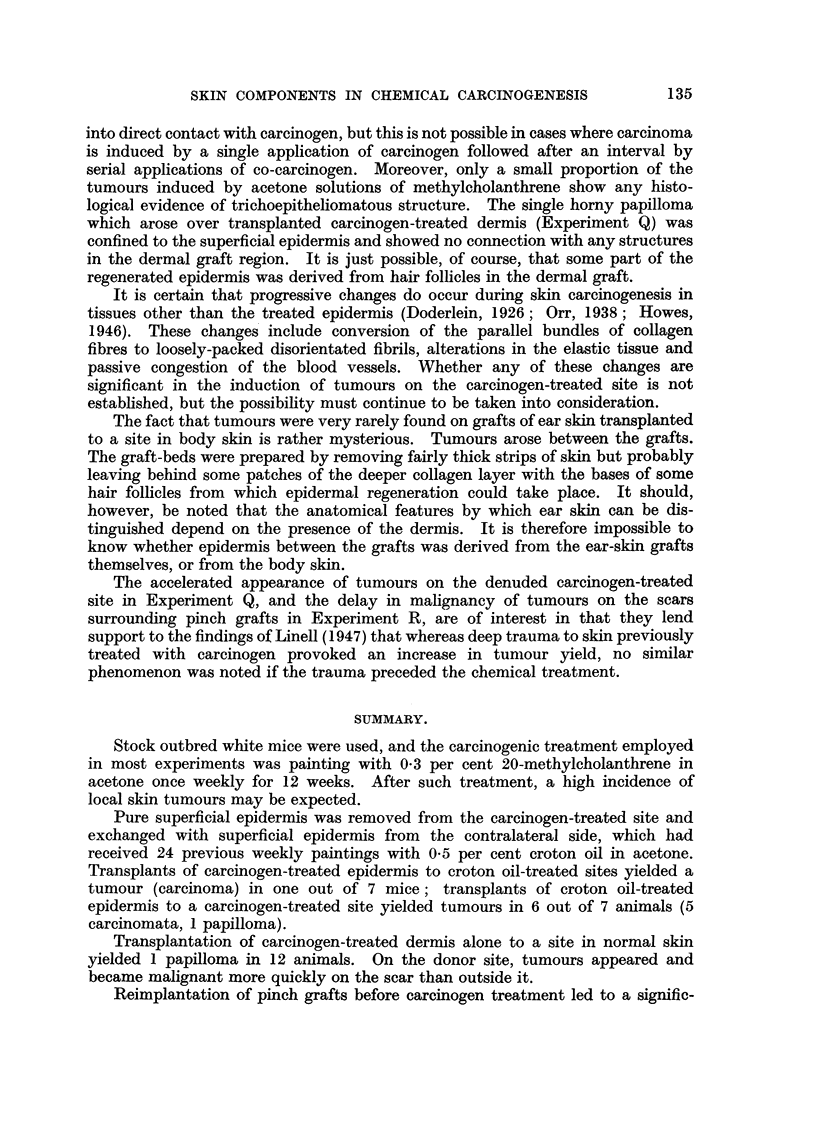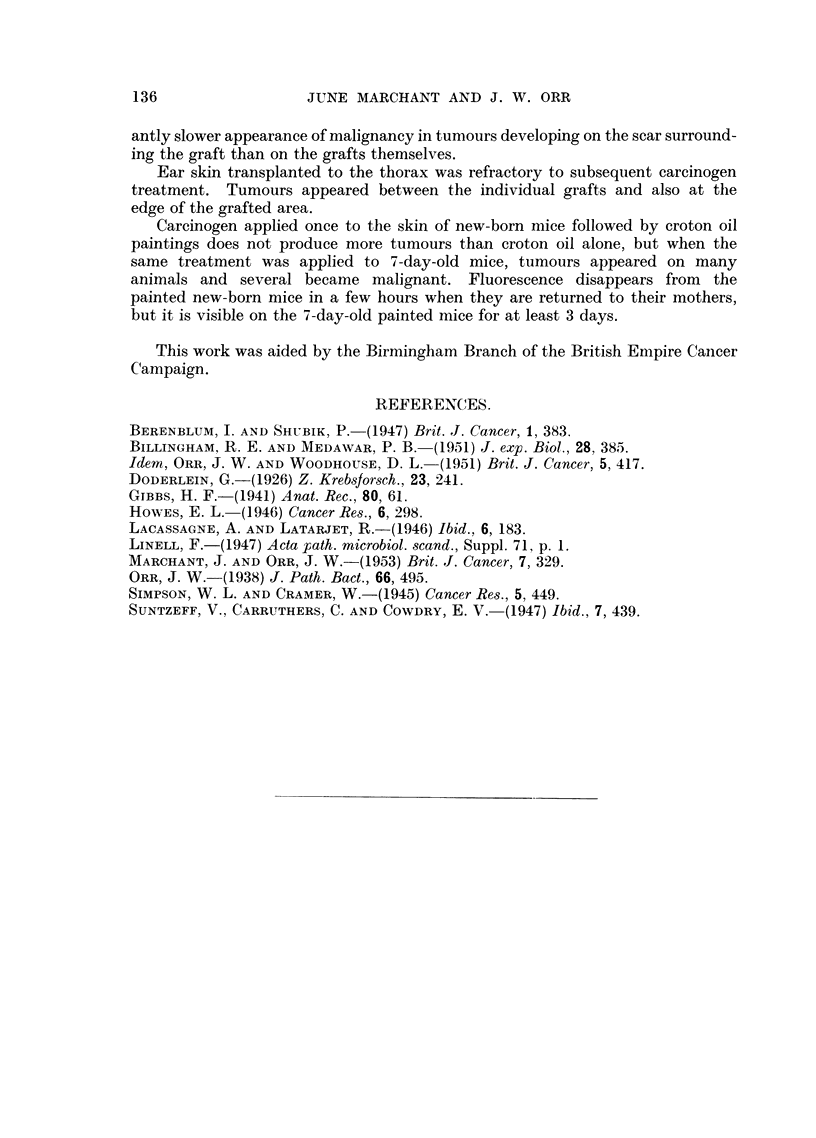# A Further Investigation of the Rôle of Skin Components in Chemical Carcinogenesis

**DOI:** 10.1038/bjc.1955.9

**Published:** 1955-03

**Authors:** June Marchant, J. W. Orr


### A FURTHER INVESTIGATION OF THE RÔLE OF SKIN COMPONENTS IN CHEMICAL CARCINOGENESIS.

JUNE MARCHANT AND J. W. ORR.

*From the Department of Pathology, University of Birmingham.*

Received for publication January 26, 1955.

PREVIOUS communications (Billingham, Orr and Woodhouse, 1951 ; Marchant and Orr, 1953) have presented results of experiments on transference of various skin layers and the effects of trauma during chemical carcinogenesis. From these experiments the following main points have emerged :

Pure epidermal or thin Thiersch grafts of carcinogen-treated skin transplanted to untreated body sites of the same mouse did not give rise to tumours. When such grafts were subsequently painted with croton oil, the number of papillomata which appeared was not significantly different from the number obtained by painting normal body skin with croton oil alone. Numerous tumours appeared on the original treated donor site. Thick Thiersch grafts and full-thickness grafts of carcinogen-treated skin yielded a small number of tumours after transplantation to sites in normal body skin. Large numbers of tumours appeared when a denuded carcinogen-treated area was left to resurface itself, or resurfaced with tail epithelium which had not received carcinogenic treatment. Reimplantation of grafts into the site from which they were cut in carcinogen-treated skin gave a number of papillomata which later regressed, but the number of persistent tumours was not increased above that of control animals.

The present experiments are further extensions of the above, and include some in which grafting procedures were carried out prior to carcinogen treatment.

### CARCINOGENIC TREATMENT.

The standard treatment employed was as described in the previous reports, namely, once weekly applications of a drop of 0·3 per cent solution of 20-methylcholanthrene in acetone for 12 weeks. The animals used were adult white mice of mixed stock and both sexes.

### OPERATIVE METHODS.

These have also been described in previous papers, grafting being done under aseptic conditions with nembutal anaesthesia. Grafts were bound in place by gauze impregnated with petroleum jelly wound firmly round the thorax of the mouse and covered with "Gypsona" plaster-impregnated bandage. The dressings were finally removed after 2 to 3 weeks.

*Experiment P ; Transplantation of carcinogen-treated pure epidermis to a recipient area cut in croton oil-painted skin and vice-versa.*

This experiment was done to find some indication whether or not tumours would arise on carcinogen-treated pure epidermis which had been transplanted to a bed whose nature had been changed by previous application of croton oil. Orr (1938) showed that croton oil was not unlike the carcinogens in its histological effects on the skin, but very much slower than them in altering the connective tissue. The changes referred to are transformation of the collagen of the dermis into a fine-fibred, non-refractile type, alterations in the texture of the elastic tissue, and passive congestion of the sub-cutis.

Seven mice were painted once weekly for 24 weeks with 0·5 per cent croton oil in acetone on the left side of the thorax and with 0·3 per cent methylcholanthrene in acetone on the right side of the thorax, during the last 12 weeks of this period. Several thin Thiersch grafts were then taken from both treated sites on each animal, removing all the obviously thickened epidermis, and the attached dermis was removed after incubation with trypsin solution (Billingham and Medawar, 1951). The pieces of pure epidermis obtained were planted back on to the site contralateral to that from which they were derived.

A persistent tumour appeared on the carcinogen-treated epidermis grafted to the croton oil-treated site of one of the 7 mice after 143 days from operation. It was clinically malignant at 162 days from the operation and histologically was a squamous carcinoma. Two small papillomas appeared on another mouse, but they regressed before the mouse died.

On the carcinogen-treated site bearing grafts of croton oil-treated epidermis, tumours appeared in 6 of the 7 mice in from 18 to 162 days from operation (mean 92 $\pm$ 26 days). Five of the tumours were clinically malignant in 40 to 162 days (mean 99 $\pm$ 23 days) from operation.

The mice survived from 94 to 234 days from operation (mean 174 $\pm$ 19 days).

The single carcinoma which arose on the carcinogen-treated epidermis transplanted to a croton oil-treated site is interesting in that this particular tumour became clinically malignant within 3 weeks of its appearance, and by the same day a tumour had appeared on the carcinogen-treated site of the same animal which was malignant from its first appearance. This might indicate that the graft of carcinogen-treated epidermis from which the tumour arose already contained some irreversibly altered cells, and that some similar irreversibly altered cells remained behind in the roots of hair follicles to give rise to a malignant tumour at exactly the same time on the original treated site.

It would seem from this experiment that treatment of skin with croton oil does not modify the dermis to such an extent that carcinogen-treated pure epidermis transplanted to it will give rise to tumours in as great number or rapid time as if left on its original site.

*Experiment Q: Transplantation of the dermis from carcinogen-treated skin to a bed cut in normal skin and natural resurfacing of the raw area on the carcinogen-treated site.*

In this experiment 12 mice were used. The epidermal surface of the carcinogen-treated site was removed as thin Thiersch grafts and a pinch graft (full thickness) was then taken from the denuded area. The panniculus carnosus muscle and fat were then trimmed off and the graft rolled in animal charcoal for identification. Such grafts would be comprised of the lower part of the collagen layer of the dermis with a few hair follicle roots. The graft was then transferred to a site prepared on the opposite untreated side of the thorax by removing a similar pinch of skin. Both sites were left to resurface themselves. The original carcinogen-treated site healed into a long scar.

A tumour appeared over the grafted dermis of one of the 12 animals after 38 days from operation and grew into a very large horn, but did not invade the deeper tissues. Histologically it consisted almost entirely of keratin. A papilloma appeared over another graft after 50 days, but it regressed after 120 days. The mice survived from 73 to 438 days after operation (mean 241 $\pm$ 30 days).

On the carcinogen-treated side of the animal 5 persistent tumours appeared on the scar and 4 outside it. The time of appearance of those on the scar was from 20 to 157 days after operation (mean 56 $\pm$ 25 days), and the time of appearance of those outside the scar was 68 to 200 days (mean 151 $\pm$ 31 days). The difference is significant $\left(\frac{\text{D}}{\text{S.E.}} = 2{\cdot}4\right)$.

Three of the tumours on the scar and 3 outside it became malignant, those on the scar after 37, 87 and 98 days (mean 74 $\pm$ 13 days). This difference is also significant $\left(\frac{\text{D}}{\text{S.E.}} = 8\right)$.

The appearance of a tumour on the grafted dermis of 1 animal in this group could be interpreted as indicating that the dermis of the carcinogen-treated skin has been altered in such a way as to be capable of inducing a tumour on regenerated epidermis. The epithelium responsible for the origin of this tumour could be derived either from the roots of hair follicles included in the graft, or from surface epithelium spreading in from the edge of the graft bed. It is impossible to choose with certainty between these two sources, but it is relevant to point out that histological examination of the remains of the grafted dermis revealed no relics of surviving follicles. A third possibility, of course, is that the dermal graft already contained at the time of operation an incipient tumour. If this were so, it is difficult to understand why it showed no evidence of infiltration.

The most interesting results of this experiment, however, are the time differences between appearance of tumours on the scar of the denuded area on the treated side of the animal and the appearance of tumours outside this scar. The significantly shorter time on the former supports the suggestion of Linell (1947) that deep trauma to a carcinogen-treated site speeds up the appearance and malignancy of tumours.

Some experiments were done to see whether grafting of skin prior to painting with a carcinogen would affect tumour production in any way.

*Experiment R: Reimplantation of a pinch graft of normal skin and subsequent painting of the graft with carcinogen.*

A pinch graft of body skin measuring about 1·5 cm. in diameter was cut from each of 18 mice. The grafts were then trimmed of fat and muscle and reimplanted, being marked round with animal charcoal. After 4 weeks, the completely healed grafts were painted with methylcholanthrene in acetone for 12 weeks. A control group of 15 ungrafted mice were painted at the same time with carcinogen.

Fourteen of the 18 mice which had skin reimplantations produced tumours, but regression occurred on 1 mouse. In another mouse the tumour was outside the grafted skin and is not counted in the following results. On the 12 remaining mice, persistent tumours first appeared after 55 to 470 days (mean 237 $\pm$ 39 days) from the first painting with carcinogen. Of these 12 tumours, 8 appeared on the grafts themselves after an average of 220 days, and 4 appeared over the scar surrounding the grafts after an average of 272 days. All the tumours arising on the grafts themselves became malignant after an average of 262 days, and 3 of the 4 on the scars did so after a significantly longer average of 364 days. The mice survived from 141 days to 638 days (mean 351 $\pm$ 32 days).

Of the 15 control mice, 13 produced tumours 107 to 320 days (mean 175 $\pm$ 19 days) after the first painting with carcinogen. Twelve of these became malignant after 142 to 380 days (mean 256 $\pm$ 19 days). The mice survived from 196 to 438 days (mean 304 $\pm$ 22 days) from first painting.

The differences in tumour production between the grafted animals and the control animals are not significant except for a delay in malignancy of the 3 tumours appearing on the scars surrounding the grafts in the experimental animals. The number of animals is, however, too small to justify inferences.

In a further group of 8 mice, an attempt was made to cut thin Thiersch grafts of normal skin and to reimplant only the pure epidermis before painting with the carcinogen. This operation is too difficult technically due to the thinness of normal mouse skin, but at least one could say that in these animals there was trauma at the dermal level prior to carcinogenic treatment.

Of these 8 mice, all produced persistent tumours on the traumatised site in from 105 to 320 days (mean 206 $\pm$ 29 days). All became malignant after 178 to 363 days (mean 280 $\pm$ 25 days) from first painting with carcinogen. The mice survived from 217 to 448 days (mean 342 $\pm$ 34 days) from first painting. The differences between these mice and the controls are not significant.

*Experiment S : Transplantation of full thickness ear skin to a bed cut in normal body skin and subsequent painting of the grafts with carcinogen.*

Fifteen mice were used. The skin of both sides of the ears was cut through 3 or 4 times from the base to the periphery and stripped with fine forceps, leaving only the central cartilage. The detached ear skin was then transplanted, in about 6 pieces, to a thoracic bed prepared by removing thick strips of body skin and dusting with animal charcoal. The grafts healed quickly, retaining the character of ear skin. The area covered by the healed grafts was 3 to 4 sq. cm. Six to 7 weeks after grafting, weekly painting with methylcholanthrene in acetone were given for 12 weeks. A group of 11 ungrafted mice was painted on the thorax at the same time to serve as controls.

Persistent tumours appeared on 14 of the 15 grafted mice. On 1 animal the tumour appeared outside the grafted area 363 days after the first painting with carcinogen. In the other 13 mice tumours arose on the grafted area after 96 to 260 days (mean 154 $\pm$ 14 days) from the first painting. Most of the mice developed several tumours some of which regressed, but at least one of them became malignant on each mouse after 119 to 320 days (mean 206 $\pm$ 17 days). Very few of the tumours arose on the actual grafts of ear skin, and only 1 of these became malignant. Most of the malignant tumours developed over the scars between two grafts, but about one-third of them arose at a slightly later average time over the zone of healing between the ear skin grafts and the normal body skin. The mice survived from 118 to 460 days (mean 260 $\pm$ 21 days) from the first painting.

There was no significant difference in tumour production between the grafted mice and the controls. In the latter persistent tumours appeared on 9 of the 11 mice after 76 to 270 days (mean 128 $\pm$ 20 days) from first painting. All of them became malignant in 91 to 314 days (mean 198 $\pm$ 22 days). The mice survived from 153 to 460 days (mean 282 $\pm$ 35 days) after the first painting.

The results of this experiment would seem to show that ear skin itself is refractory to the production of tumours by methylcholanthrene in acetone when transplanted to a site in body skin. (We had previously found this to be so with ear skin *in situ*). Most of the tumours arose between the pieces of ear skin.

*Experiment T: Reimplantation of thin Thiersch grafts of normal skin and subsequent painting with croton oil.*

This experiment was done as a control for Experiment J (Marchant and Orr, 1953) in which pure epidermis was transplanted from a carcinogen-treated site to a site in untreated skin and then painted weekly with croton oil for the remainder of life. In addition to a small number of papillomata, such as are obtained by croton oil painting alone, a single carcinoma occurred on such epidermis in one of 37 mice. It was considered desirable to know whether modification of the dermis of normal skin as a result of the grafting operation would affect the tumours yielded by subsequent croton oil painting.

Since it is extremely difficult to obtain thin Thiersch grafts or pure epidermal grafts from normal body skin in the mouse, the skin was treated once or twice before grafting with croton oil to induce hyperplasia. Thin Thiersch grafts were then cut from such skin in 24 mice and reimplanted, being marked with animal charcoal. After about 4 weeks, weekly paintings with 0·5 per cent croton oil in acetone were commenced. A control group of 9 animals received similar paintings, but no grafting operation.

Of the 20 grafted animals, 4 developed papillomas after 272 to 418 days (mean 344 days). Only 1 of these was actually on a graft, the other 3 being on surrounding skin. One of the latter regressed in 40 days. The survival of these mice was from 177 to 559 days (mean 403 $\pm$ 23 days).

Of the 9 control mice, only 1 developed a papilloma after 340 days. The survival of these mice was from 138 to 454 days (mean 239 $\pm$ 42 days).

We include here the final results for the control animals in the previous Experiment J (Marchant and Orr, 1953). In this experiment 36 mice received once weekly paintings of croton oil without any previous treatment. Persistent tumours appeared on 7 of them in 247 to 540 days (mean 337 $\pm$ 41 days) from first painting. None became malignant. The mice survived from 212 to 676 days (mean 388 $\pm$ 23 days).

It will thus be seen that there was no significant difference between the two groups of Experiment T and the control group of Experiment J with regard to tumour yield or time of appearance of tumours. It may be concluded that preliminary Thiersch grafting does not modify the dermis in such a way as to affect the yield of tumours by croton oil painting.

The general trend of the results of these and the previously reported experiments has been to suggest that the tumours which arise in carcinogen-treated skin are not determined by changes in the superficial epidermis itself, and that the major precancerous factors are located in the deeper layers. Reasons have been given for attaching importance to the connective tissue and other stromal elements, but there still remains the possibilty that the effective source of the tumours and site of action of the carcinogen may be the epithelium of the hair follicles. It occurred to us that by using the technique of co-carcinogenesis (Berenblum and Shubik, 1947) and new born mice without penetrated hairs or sebaceous glands, an answer might be obtained to this question.

*Experiment U* : *Single painting with carcinogen on day of birth, or on seventh day after birth followed by croton oil to develop the tumours.*

Two groups of mice were used in this experiment. The first, consisting of 9 animals, were given one painting of methylcholanthrene in acetone on the side of the thorax on the day of birth (before hair follicles had developed—Gibbs, 1941) and immediately returned to their mothers. The second group of 15 animals received a similar painting on the 7th day after birth (when hair follicles had developed) and were returned to their mothers. When the mice were 4 weeks old, weekly paintings of 0·5 per cent croton oil in acetone on the same site were commenced.

Of the 9 mice painted on the day of birth, 4 developed persistent tumours. One of these was a mammary tumour which arose 520 days after the methylcholanthrene painting. The other 3 were papillomas arising after 275, 368 and 464 days (mean 369 days). None of these papillomas became malignant. The mice survived from 193 to 568 days (mean 489 $\pm$ 44 days).

Of the 15 mice painted with carcinogen on the 7th day after birth, 10 developed tumours after 89 to 460 days (mean 192 $\pm$ 35 days). Six of the tumours became malignant after 280 to 490 days (mean 348 $\pm$ 32 days). The appearance of tumours on these mice was significantly quicker than in the mice painted with carcinogen on the day of birth. Their lives were significantly shorter, the survival being from 130 to 518 days (mean 358 $\pm$ 33 days).

It will be seen that tumours developed much more infrequently and slowly on the skin which received its application of carcinogen before the development of hair follicles than on the skin to which carcinogen was applied after hair follicles had developed. In fact, the time taken by the former is no different from the time taken by croton oil alone to elicit tumours in the control mice for Experiment J. In other words, carcinogen applied to mouse body skin on the day of birth is ineffective in producing carcinomas. This could be interpreted to mean that the presence of hair follicles in the skin is required, possibly as a mode of entry and storage of the carcinogen, in order that tumours should be elicited. But it is possible that the mothers of the new-born mice lick off all the applied carcinogen before it has time to act. In order to test this point, some new-born and some 7-day old mice were painted with methylcholanthrene, returned to their mothers, and observed at intervals under an ultra-violet lamp to see how long the skin remained fluorescent in each case. The following results were obtained : Fluorescence was considerably faded from the newborn mice after 4 hours and completely disappeared after 24 hours. One baby which was kept in the warm away from the mother was still alive and showed bright fluorescence after 24 hours. The 7-day old mice were still brightly fluorescent after 24 hours, and faintly so after 3 days.

### DISCUSSION.

The results of Experiment U in which no malignant tumours were obtained after a single application of carcinogen to the skin of new-born mice, followed by painting with croton oil, confirm the previous experiments of Suntzeff, Carruthers and Cowdry (1947). They obtained no tumours after application of 0·6 per cent methylcholanthrene in benzene to new-born mice and considered failure might be due to the thickness of the epidermis (5 to 7 cells) preventing penetration of the carcinogen, or to the scarcity of penetrated hair follicles and the lack of sebaceous glands. We have also considered the fact that the carcinogen might be licked off by the mothers very rapidly, and have found it does disappear during the first 24 hours. The thickness can probably be discounted, since tumours will develop after carcinogen application to epidermis rendered hyperplastic by such agents as croton oil.

The necessity of hair follicles in the mechanism of skin carcinogenesis is suggested by the experiments of Lacassagne and Latarjet (1946). They gave repeated applications of methylcholanthrene in acetone to ultra-violet burns on the skin of new-born and adult mice. When the applications were begun 10 days after the burning (at a time when there had been some regeneration of hairs and sebaceous glands) tumours appeared on the scar. If the applications were begun on the day following the burn, healing was unimpaired, but no hair follicles appeared on the scar and nor did any tumours. From our almost invariable failure to obtain tumours on grafts of pure carcinogen-treated epidermis transplanted to untreated sites, we can say with some confidence that, at a stage when the carcinogen-treated site is capable of epithelial neoplasia, this potentiality is not an intrinsic property of the cells of the superficial epidermis. Therefore, if the carcinogenesis is determined by a primary intrinsic change in epithelial cells alone, the parent cells of the tumour might be those of the hair follicles or sebaceous glands.

There is no doubt that the hair follicles and sebaceous glands represent an important mode of entry of the carcinogen into the skin. Simpson and Cramer (1945) for example, were able to show by fluorescence studies, that methylcholanthrene reaches the sebaceous glands almost immediately after superficial application. At a later stage some of it is found in the dermal connective tissues, but fluorescence persists in the sebaceous glands for a considerable time. The cells of the hair follicle are thus exposed to the direct action of carcinogen for a greater time and in greater concentration than those of the superficial epidermis, and it is impossible to exclude them as the actual site of carcinogenesis.

There are, however, some difficulties in accepting the hair follicles as the source of most of the tumours. In the early stages of chemical carcinogenesis with methylcholanthrene, complete epilation of the treated area of skin occurs after one or two weeks, and histological examination at this stage shows disappearance of most or even all of the hair follicles. Regeneration subsequently takes place, and with continued treatment there follow cycles of epilation and regeneration (Orr, 1938). Thus the hair follicles which originally receive the carcinogen do not survive to the time when tumours may be expected to develop. In the case of experiments where a series of applications of carcinogen is made, there are of course opportunities for the later generations of hair follicles to come into direct contact with carcinogen, but this is not possible in cases where carcinoma is induced by a single application of carcinogen followed after an interval by serial applications of co-carcinogen. Moreover, only a small proportion of the tumours induced by acetone solutions of methylcholanthrene show any histological evidence of trichoepitheliomatous structure. The single horny papilloma which arose over transplanted carcinogen-treated dermis (Experiment Q) was confined to the superficial epidermis and showed no connection with any structures in the dermal graft region. It is just possible, of course, that some part of the regenerated epidermis was derived from hair follicles in the dermal graft.

It is certain that progressive changes do occur during skin carcinogenesis in tissues other than the treated epidermis (Doderlein, 1926; Orr, 1938; Howes, 1946). These changes include conversion of the parallel bundles of collagen fibres to loosely-packed disorientated fibrils, alterations in the elastic tissue and passive congestion of the blood vessels. Whether any of these changes are significant in the induction of tumours on the carcinogen-treated site is not established, but the possibility must continue to be taken into consideration.

The fact that tumours were very rarely found on grafts of ear skin transplanted to a site in body skin is rather mysterious. Tumours arose between the grafts. The graft-beds were prepared by removing fairly thick strips of skin but probably leaving behind some patches of the deeper collagen layer with the bases of some hair follicles from which epidermal regeneration could take place. It should, however, be noted that the anatomical features by which ear skin can be distinguished depend on the presence of the dermis. It is therefore impossible to know whether epidermis between the grafts was derived from the ear-skin grafts themselves, or from the body skin.

The accelerated appearance of tumours on the denuded carcinogen-treated site in Experiment Q, and the delay in malignancy of tumours on the scars surrounding pinch grafts in Experiment R, are of interest in that they lend support to the findings of Linell (1947) that whereas deep trauma to skin previously treated with carcinogen provoked an increase in tumour yield, no similar phenomenon was noted if the trauma preceded the chemical treatment.

### SUMMARY.

Stock outbred white mice were used, and the carcinogenic treatment employed in most experiments was painting with 0·3 per cent 20-methylcholanthrene in acetone once weekly for 12 weeks. After such treatment, a high incidence of local skin tumours may be expected.

Pure superficial epidermis was removed from the carcinogen-treated site and exchanged with superficial epidermis from the contralateral side, which had received 24 previous weekly paintings with 0·5 per cent croton oil in acetone. Transplants of carcinogen-treated epidermis to croton oil-treated sites yielded a tumour (carcinoma) in one out of 7 mice; transplants of croton oil-treated epidermis to a carcinogen-treated site yielded tumours in 6 out of 7 animals (5 carcinomata, 1 papilloma).

Transplantation of carcinogen-treated dermis alone to a site in normal skin yielded 1 papilloma in 12 animals. On the donor site, tumours appeared and became malignant more quickly on the scar than outside it.

Reimplantation of pinch grafts before carcinogen treatment led to a significantly slower appearance of malignancy in tumours developing on the scar surrounding the graft than on the grafts themselves.

Ear skin transplanted to the thorax was refractory to subsequent carcinogen treatment. Tumours appeared between the individual grafts and also at the edge of the grafted area.

Carcinogen applied once to the skin of new-born mice followed by croton oil paintings does not produce more tumours than croton oil alone, but when the same treatment was applied to 7-day-old mice, tumours appeared on many animals and several became malignant. Fluorescence disappears from the painted new-born mice in a few hours when they are returned to their mothers, but it is visible on the 7-day-old painted mice for at least 3 days.

This work was aided by the Birmingham Branch of the British Empire Cancer Campaign.

### REFERENCES.

Berenblum, I. and Shubik, P.—(1947) *Brit. J. Cancer*, **1**, 383.
Billingham, R. E. and Medawar, P. B.—(1951) *J. exp. Biol.*, **28**, 385.
*Idem*, Orr, J. W. and Woodhouse, D. L.—(1951) *Brit. J. Cancer*, **5**, 417.
Doderlein, G.—(1926) *Z. Krebsforsch.*, **23**, 241.
Gibbs, H. F.—(1941) *Anat. Rec.*, **80**, 61.
Howes, E. L.—(1946) *Cancer Res.*, **6**, 298.
Lacassagne, A. and Latarjet, R.—(1946) *Ibid.*, **6**, 183.
Linell, F.—(1947) *Acta path. microbiol. scand.*, Suppl. 71, p. 1.
Marchant, J. and Orr, J. W.—(1953) *Brit. J. Cancer*, **7**, 329.
Orr, J. W.—(1938) *J. Path. Bact.*, **66**, 495.
Simpson, W. L. and Cramer, W.—(1945) *Cancer Res.*, **5**, 449.
Suntzeff, V., Carruthers, C. and Cowdry, E. V.—(1947) *Ibid.*, **7**, 439.